# Urinary Equol Production Capacity, Dietary Habits, and Premenstrual Symptom Severity in Healthy Young Japanese Women

**DOI:** 10.3390/metabo16010055

**Published:** 2026-01-08

**Authors:** Nanae Kada-Kondo, Natsuka Kimura, Kurea Isobe, Akari Kaida, Saki Ota, Akari Fujita, Yuu Haraki, Ryozo Nagai, Kenichi Aizawa

**Affiliations:** 1 Department of Food Science, Faculty of Home Economics, Otsuma Women’s University, 12, Sanbancho, Chiyoda 102-8357, Tokyo, Japan; 2 Department of Nutrition and Dietetics, Faculty of Home Economics, Kamakura Women’s University, 6-1-3, Ofuna, Kamakura 247-8512, Kanagawa, Japan; 3 Department of Translational Research, Clinical Research Center, Jichi Medical University Hospital, 3311-1, Yakushiji, Shimotsuke 329-0498, Tochigi, Japan; 4 Jichi Medical University, 3311-1, Yakushiji, Shimotsuke 329-0498, Tochigi, Japan

**Keywords:** equol, soy isoflavones, premenstrual syndrome, dietary habits, urinary metabolites, gut microbiota, LC–MS/MS, women’s health, metabolomics

## Abstract

**Background/Objectives**: Equol, a gut microbial metabolite of the soy isoflavone, daidzein, is associated with estrogenic activity and potential benefits for women’s health. While equol production depends on individual gut microbial composition, its dietary and clinical correlates in young women remain incompletely characterized. This study explored the relationship between urinary equol production, dietary habits, and premenstrual symptom severity in healthy university-aged women. **Methods**: We conducted a cross-sectional study of 41 Japanese women, aged 19–20 years. Urinary equol was measured using a validated liquid chromatography–tandem mass spectrometry (LC–MS/MS) method, following enzymatic hydrolysis. Participants were classified as either equol producers or non-producers, based on urinary concentration thresholds. Dietary intake was evaluated using a dietary questionnaire focused on soy products and dietary fiber sources. Premenstrual symptoms were assessed using a standardized Japanese questionnaire for premenstrual syndrome and premenstrual dysphoric disorder. **Results**: Twelve percent of participants were classified as equol producers. Compared with non-producers, equol producers reported higher consumption of pumpkin, soybean sprouts, and green tea. Among non-producers, higher consumption of certain vegetables and fiber-rich foods, including broccoli, pickled radish, konjac, and konjac jelly, was associated with greater premenstrual symptom severity, whereas such associations were not observed among equol producers. The analytical method demonstrated high sensitivity and reproducibility for urinary equol measurement. **Conclusions**: These findings suggest that equol production status may be associated with distinct dietary patterns and with differences in the relationship between food intake and premenstrual symptom severity in young women. Although the cross-sectional design and limited sample size preclude causal inference, these findings suggest that urinary equol is a promising candidate biomarker for future research on diet-related modulation of premenstrual symptoms.

## 1. Introduction

Equol is a metabolite produced by intestinal biotransformation of daidzein, a soy isoflavone, by specific gut microbes. Owing to its structural similarity to estrogen, equol is believed to possess estrogen-like biological activities and has been associated with attenuation of various conditions influenced by female sex hormones [1]. Recent research has highlighted the therapeutic potential of equol, particularly in management of menopausal symptoms. Its beneficial effects include suppression of bone resorption and maintenance of bone mineral density [2,3,4], reduction of vasomotor symptoms such as hot flashes, alleviation of musculoskeletal discomfort such as neck and shoulder stiffness [2,3,4], and improvement in glucose metabolism, evidenced by lowered HbA1c levels [5,6]. Notably, equol was officially recognized as a dietary supplement for menopausal symptom management in the 2017 edition of a clinical guidebook for physicians in Japan [7].

In Japan, it has been reported that equol production capacity is generally lower among younger women [8]. A number of studies have examined whether continuous consumption of soy products could enhance endogenous equol production. Furthermore, it has been suggested that even individuals lacking gut bacteria necessary to produce equol may benefit from dietary intake of equol-containing supplements [9,10].

Recently, attention has turned to a possible link between equol and premenstrual symptoms, particularly premenstrual syndrome (PMS) and premenstrual dysphoric disorder (PMDD) [11,12]. PMS encompasses a range of mental and physical symptoms that arise approximately one week prior to menstruation and typically resolve with the onset of bleeding. Psychological symptoms may include anxiety, irritability, fatigue, and reduced concentration, whereas physical symptoms often involve lower abdominal pain, headaches, insomnia, and bloating. When psychological symptoms such as irritability and anxiety are particularly pronounced, a diagnosis of PMDD may be warranted [11,12,13]. These premenstrual symptoms are believed to result from fluctuations in sex hormone levels between ovulation and menstruation, along with altered neurotransmitter activity and autonomic nervous system dysregulation [14].

Emerging data indicate that oxidative stress and neuroinflammation may contribute to the pathophysiology of PMS/PMDD. Equol, with its established antioxidant properties, may mitigate oxidative stress by scavenging reactive oxygen species and supporting endogenous antioxidation [1,15]. However, few studies have examined equol production capacity specifically in young women, as most existing research has focused on postmenopausal populations or soy isoflavone intake itself, and its association with premenstrual symptoms and relevant dietary factors remains poorly understood.

While recent reports indicate that equol production is often absent or reduced in women aged 20 to 45 undergoing treatment for PMS/PMDD, data on younger populations remain scarce [10]. Given the emerging interest in equol as a modulator of oxidative stress and hormonal balance, the present study sought to clarify the association between equol-producing capacity and the presence or absence of PMS/PMDD symptoms in a cohort of young women (aged 19–20 years), with additional emphasis on dietary patterns potentially involved in symptom modulation.

## 2. Materials and Methods

This study enrolled 42 healthy Japanese women aged 19 to 20 years who gave informed consent to participate. To examine the relationship between equol-producing capacity, dietary habits, and premenstrual symptomatology, we conducted comprehensive surveys encompassing both dietary intake and PMS/PMDD status, as detailed below.

For dietary assessment, we developed a customized dietary questionnaire based on a semi-quantitative dietary questionnaire. Particular emphasis was placed on food items associated with equol production, such as dietary fiber, soy products, and seaweed [16,17]. The questionnaire included 30 items, including nine soy-based foods, various vegetables, mushrooms, seaweed, caffeine-rich beverages, alcohol, konjac (a traditional root-derived food), and konjac jelly (a fruit-flavored snack). For each item, both frequency and quantity of intake were recorded.

Intake frequency was categorized into six levels: “hardly ever”, “once a month”, “1–2 times per week”, “3–4 times per week”, “5–6 times per week”, and “once a day or more”. Intake quantity was classified as “0.5×”, “0.8×”, “equal to”, “1.5×”, “≥2× the standard amount”, or “not consumed”. For konjac jelly, an additional category of “three times or more” was incorporated to account for large consumption volumes.

Of the 42 participants, one did not complete the dietary questionnaire and was excluded from analyses. Intake frequency was dichotomized as “high” if the item in question was consumed ≥1–2 times per week, or “low” otherwise. For intake quantity, values exceeding the standard portion size defined in the Japanese Dietary Reference Intakes were categorized as “consumed”, while lower values were considered “not consumed [18]”.

For assessment of PMS/PMDD, diagnostic classification was performed in accordance with established criteria and standardized evaluation forms published by the Japan Society of Obstetrics and Gynecology [11]. Participants were stratified into three categories, none/mild, moderate, or severe, based on symptom severity and further classified according to their equol-producing status [11] (Supplementary Table S1).

To determine equol production capacity, participants ingested soy products, e.g., one serving of natto or 200 mL of soy milk, the evening before urine collection. First-morning urine samples were collected. Urine samples were divided into two groups: one for equol measurement by liquid chromatography–tandem mass spectrometry (LC–MS/MS) and the other for creatinine measurement using an enzymatic method. Urine creatinine concentrations were quantified at an external testing facility, following the facility’s clinical practice guidelines. Equol concentrations measured by LC-MS/MS were normalized for creatinine to correct for urine concentrations. Equol-producing status was classified using the cutoff value (1.0 µM) applied [19].

### 2.1. Chemicals and Reagents

(±)-Equol (>98.0%) (EQ) was purchased from Tokyo Chemical Industry (Tokyo, Japan). (R,S)-Equol-d4 (>95%) (EQ-d4) was purchased from Toronto Research Chemicals (Toronto, ON, Canada). β-Glucuronidase from *Helix pomatia* (100,000 U/mL), sulfatase (type HP-2) from *H. pomatia* (<7500 U/mL), and dimethyl sulfoxide (>99.5%) (DMSO) were purchased from Sigma-Aldrich (St. Louis, MO, USA). Acetic acid glacial (99.9985%) was purchased from Thermo Fisher Scientific (Waltham, MA, USA). Sodium acetate trihydrate (Guaranteed Reagent), formic acid (LCMS grade), acetonitrile (LCMS grade), Methanol (LCMS grade) and ultra-pure water (LCMS grade) were purchased from FUJIFILM Wako Pure Chemical (Osaka, Japan).

### 2.2. Preparation of Reagents

A stock standard solution (EQ) was prepared at 100 mM in DMSO and stored at −80 °C. The stock solution of internal standards (EQ-d4) was prepared at 1 mg/mL in methanol and stored at −80 °C. A 1 mM acetonitrile solution of EQ was prepared for each measurement as a standard solution. This was diluted stepwise from 0.05 µM to 500 µM with 50% acetonitrile to prepare calibration curve samples. A 0.5 µg/mL methanol solution of EQ-d4 was prepared for each measurement as an internal standard solution. 0.1 M acetate buffer (pH 4.7) was prepared by dissolving 2.87 mL of glacial acetic acid and 6.8 g of sodium acetate trihydrate in 1 L of water. After pH adjustment, it was stored in a cool, dark place and prepared as needed. The enzyme solution for deglucuronidation (gHP) was prepared by dissolving *H. pomatia* β-glucuronidase (100,000 U/mL) in 0.1 M acetate buffer (pH 4.7) at a concentration of 1200 U/mL. The enzyme solution for desulfation (sHP) was prepared by dissolving sulfatase (≥10,000 U/g solid) derived from *H. pomatia* in 0.1 M acetate buffer (pH 4.7) at a concentration of 1 mg/mL (10 U/mL).

### 2.3. Preparation of Standard Solution and Human Urine Samples

50 µL of human urine or 50 µL of 50% acetonitrile standard solution were mixed with 10 µL of internal standard solution (0.5 µg/mL-MeOH), 100 µL of gHP 1200 U/mL, and 100 µL of sHP 10 U/mL, and incubated at 45 °C for 4 h or more. After incubation, 800 µL of acetonitrile were added to the sample, vortexed, and centrifuged (12,000 rpm, 10 min, 4 °C). After centrifugation, 150 µL of supernatant were transferred to another tube, concentrated, and dried using a centrifugal concentrator. In total, 100 µL of 0.1% formic acid–water were added to the dried residue, mixed, and centrifuged (12,000 rpm, 10 min, 4 °C). Supernatant was transferred to an analytical vial for LCMS analysis.

### 2.4. Instrumentation and Analytical Conditions

All analyte concentrations were analyzed by LC-MS/MS (LCMS-8060 NX System, Shimadzu, Kyoto, Japan). For LC analyses, a Shim-pack Scepter C_18_ analytical column (50 × 2.1 mm 2 µm) was used. The column oven and autosampler were set to 50 °C and 4 °C, respectively. Mobile phase A was 0.1% formic acid–water, and mobile phase B was acetonitrile. The flow rate was set to 0.25 mL/min, and the injection volume was 2 μL. The gradient program was as follows: 0 to 0.5 min, %B = 10; 0.5 to 3.8 min, %B = 10 to 85% gradient; 3.81 min, %B = 10; 3.81 to 5.5 min, %B = 98%; 5.51 to 7 min, %B = 10%.

Analytes were detected in ESI-positive mode. MS/MS conditions were as follows: Nebulizer gas flow (3 L/min), heating gas flow (10 L/min), interface temperature (400 °C), desolvation temperature (650 °C), heat block temperature (400 °C), and drying gas flow (10 L/min). Collision energies were 13 V for EQ, 27 V for EQ-d4. EQ and EQ-d4 were observed at *m*/*z* 243.2 > 123.1 and 247.1 > 125, respectively. EQ concentration of samples was quantified by the area ratio with the internal standard reagent added to the samples. All urinary equol values were normalized to creatinine.

Statistical analyses were performed to compare PMS/PMDD severity and dietary patterns between equol producers and non-producers. Analytical methods included *t*-tests and chi-square tests. A significance level of *p* < 0.05 was applied throughout. Univariable comparisons were performed using *t*-tests or chi-square tests, as appropriate. Subsequently, multivariable logistic regression analyses were conducted to adjust for potential confounding factors.

This study was conducted with approval of the ethics committee of Kamakura Women’s University (approval codes: Kamarin-24014; approval dates: 31 July 2024).

## 3. Results

### 3.1. Method Development

In the standard procedure for quantitative analysis of phytoestrogens in body fluids, i.e., urine, serum, plasma, phytoestrogen conjugates are usually converted to aglycones by enzymatic hydrolysis before sample extraction. Taylor et al. [20] reported various hydrolytic conditions that have been used for plasma and urine samples. In this study, enzymatic hydrolytic conditions of Taylor et al. [20] were adopted, and each validation was performed after first examining incubation conditions sufficient for enzymatic hydrolysis. Enzyme was added to blank samples to confirm the presence of enzyme contamination, and lower limits of quantification of target compounds were determined (Supplementary Figure S1). The LC-MS/MS method was validated in accordance with Bioanalytical Method Validation (BMV) guidelines issued by the Food and Drug Administration, USA (FDA) [21].

### 3.2. Method Validation

The analytical method employed LC–MS/MS using a validated protocol for quantification of urinary equol concentrations. This assay demonstrated high sensitivity and specificity.

The calibration curve consisted of a zero sample (internal standard addition blank) and 5 calibration samples including the lower limit of quantification (LLOQ), from which the regression equation and correlation coefficient were calculated. Precision of all quality control (QC) concentrations from the regression equation was calculated using the average value of three runs on different measurement days. Precision was within ±20% of the theoretical value for the LLOQ and ±15% of the theoretical value for non-LLOQ concentrations. All points of the calibration curve met these criteria (Supplementary Figure S2).

Intra-day precision and accuracy were assessed by analyzing five QC samples at three concentrations in a batch in triplicate. Accuracy was calculated as a percentage of the measured concentration relative to the nominal concentration. Accuracy was within ±10% at all concentrations. To assess precision, the coefficient of variation (CV) between measured concentrations was calculated. Precision was within ±15% at all concentrations tested (Supplementary Table S2). Inter-day precision was determined by analyzing seven samples (three QC samples and four urine samples) in three independent runs. EQ was below the lower limit of quantitation for two of the four urine samples in all runs. Precision was assessed by calculating the coefficient of variation between measured concentrations. Inter-day precision was within ±15% for samples with detectable EQ concentrations (Supplementary Table S3). Extract stability was evaluated for seven samples (three QC samples and four urine samples). After enzymatic digestion, samples were deproteinated by adding acetonitrile. The residue obtained by evaporation of acetonitrile from deproteinated supernatant was reconstituted in water containing 0.1% formic acid and stored in an autosampler (4 °C) until analysis. Stability of the supernatant was measured after 0, 12, and 24 h. Stability was assessed by area ratio, and a CV% of 15% or less was considered stable. Based on these results, all analyses were performed within 24 h (Supplementary Table S4).

Characteristics of the study population are summarized in Table 1. Equol-producing status was classified using the cutoff value applied in the Japan Nurses’ Health Study [19], where this threshold was statistically determined as optimal for classifying equol producing status in observational settings.

**Figure 1:**
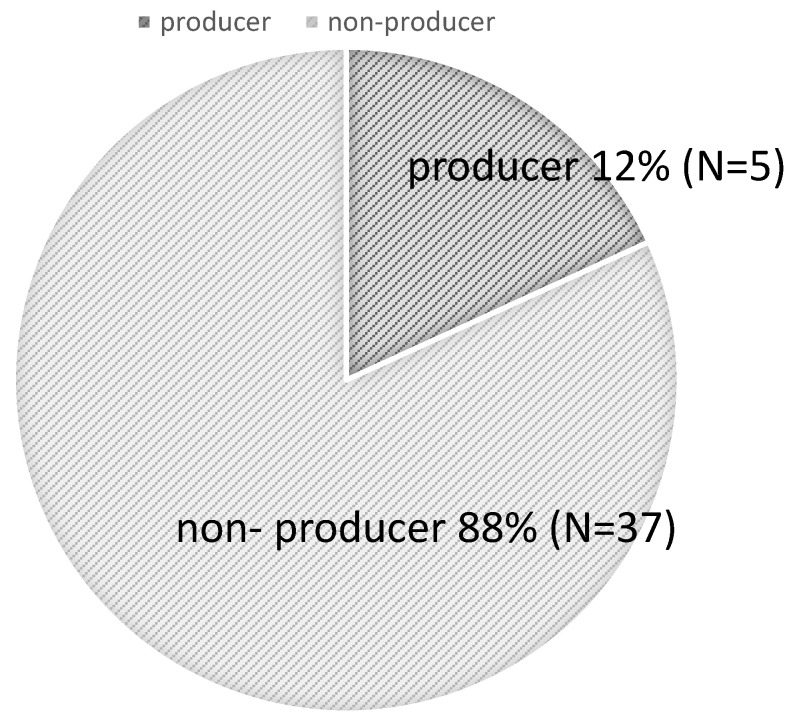
Proportion of Equol producers in the Study Population.

Participants with urinary equol levels of ≥1.0 μM were classified as producers, and those with levels < 1.0 μM as non-producers. Among the 41 participants, the proportion of equol producers was 12.0%, while non-producers accounted for 88.0% (Figure 1). This frequency of equol producers is slightly lower than in previous reports [8], which estimated the prevalence of equol producers at 20%, suggesting that the capacity for equol production may be even lower among women aged 19–20 years.

Dietary patterns revealed that equol producers more frequently consumed pumpkin and green tea (unsweetened) compared to non-producers (Table 2). Furthermore, quantities of soybean sprouts and pumpkin exceeding the recommended amounts were significantly higher among equol producers (Table 3). These findings suggest that both the frequency and quantity of pumpkin intake, in particular, may support equol production.

Numbers of participants stratified by equol-producing status and PMS/PMDD severity are shown in Table 4.

**Figure 2:**
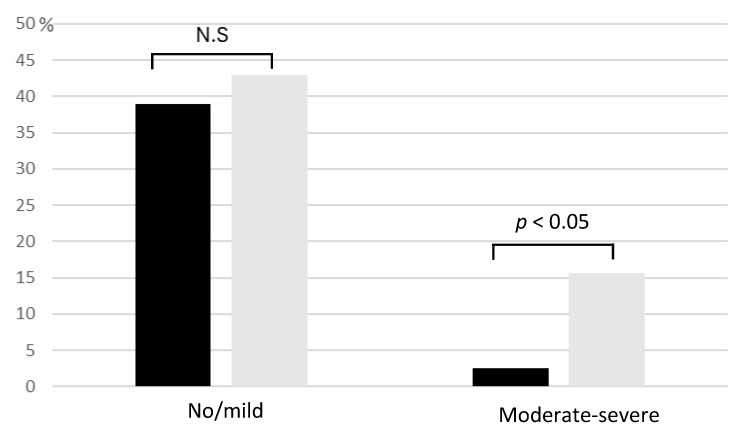
Distribution of Premenstrual Symptoms in Relation to Equol Production Status. Black bars indicate equol producers, and gray bars represent non-producers. While equol production status showed no significant difference among participants with no or mild PMS/PMDD symptoms, more non-producers were found among those experiencing moderate to severe symptoms. Cases without statistical significance were indicated as N.S. (not significant).

No significant association was observed between equol production and individual symptoms of the PMS/PMDD questionnaire. However, when categorizing participants by symptom severity, a notable trend emerged. While no significant difference in equol production was evident among those with no or mild symptoms, individuals experiencing moderate to severe symptoms included significantly fewer equol producers (Figure 2).

Subsequently, we examined the relationship between dietary habits and PMS/PMDD symptom severity in equol producers and non-producers. Equol-producing capacity was included as the main explanatory variable and age and PMS severity were employed as covariates in a multivariable logistic regression model. The association between equol-producing capacity and outcome was not substantially attenuated (Table 5).

Among equol producers, no consistent associations were observed between overall dietary intake patterns and symptom severity; however, a higher frequency of black tea consumption was associated with greater symptom severity (Table 6 and Table 7). In contrast, in the non-producer group, increased symptom severity was significantly associated with higher intake of broccoli and greater consumption of pickled radish, konjac, and konjac jelly (Table 8 and Table 9). These findings indicate that in the absence of equol production capacity, certain dietary components may influence the severity of PMS/PMDD symptoms.

## 4. Discussion

The proportion of equol producers among 19–20-year-old participants in this study was lower than that reported for individuals in their 20s [8,22]. This finding is consistent with previous reports suggesting that equol-producing capacity may continue to evolve during early adulthood. The difference observed in this study between equol-producing capacity in individuals aged 19–20 years and that reported for people in their 20s overall may be attributable not only to differences in the degree of dietary westernization, e.g., reduced intake of soy products and fermented foods [8], but also to the ongoing maturation and stabilization of the gut microbiota during this life stage. Equol-producing bacteria are thought to establish more readily in a sufficiently mature and stable gut environment; however, equol-producing capacity is not a fixed trait and may be reversibly acquired or lost in response to dietary and other environmental factors. Numerous studies have shown that the gut microbiota continues to change throughout life [23], and even during adulthood, age-related changes in microbial composition and diversity have been documented [24]. Large-scale cohort analyses have further supported age-associated remodeling of the gut microbiota throughout adulthood [25]. Taken together, these observations provide a plausible biological context for the relatively low prevalence of equol producers observed in this cohort.

First, the limited proportion of equol producers among women in their early twenties resulted in a small number of participants with detectable equol-producing capacity. This imbalance likely reduced the statistical power of subgroup analyses and should be considered when interpreting the results. Accordingly, the present study was conducted as an exploratory, hypothesis-generating investigation, and its findings should be interpreted in this context.

Dietary intake was assessed using a semi-quantitative questionnaire designed to capture relative intake patterns rather than precise nutrient amounts. Although this method is subject to recall bias and may result in non-differential misclassification, it is considered appropriate, given the exploratory nature of this study. Future studies using more rigorous dietary assessment methods and longitudinal or interventional designs are needed to confirm these findings.

When equol-producing capacity was included as the main explanatory variable and age and PMS severity were employed as covariates in a multivariable logistic regression model, the association between equol-producing capacity and outcome was not substantially attenuated. This suggests that the observed association cannot be fully explained by differences in age or baseline PMS severity alone. Although residual confounding cannot be excluded, these results raise the possibility that equol-producing capacity may be associated with PMS-related outcomes beyond these factors. However, given the cross-sectional design and limited sample size, these findings should not be interpreted as evidence of causality.

Overall, while the limited sample size precludes definitive conclusions, the present findings provide preliminary support for a relationship between equol-producing capacity and clinical features of PMS. Further longitudinal and interventional studies with larger sample sizes are required to confirm these associations and to clarify their clinical relevance.

The relationship between equol-producing capacity and dietary habits suggests that equol producers consume more pumpkin and green tea, as well as greater quantities of soybean sprouts. Although no previous studies have directly reported an association between pumpkin intake and equol-producing capacity, dietary fiber is essential in maintaining the intestinal environment and is considered important for equol-producing bacteria [26]. The Western pumpkin examined in this study contains approximately 0.9 g of soluble dietary fiber per 100 g [27], which may promote fermentation by gut microbiota. Experimental studies using ovariectomized mice have shown that soluble fibers such as polydextrose and raffinose increase equol production [28]. Resistant starch also reportedly enhances equol biosynthesis [29]. Accordingly, the observed association between pumpkin intake and equol production may reflect the combined effects of soluble fiber and resistant starch, although this interpretation remains speculative. Therefore, because pumpkin is rich in both soluble fiber and resistant starch [30], its consumption may be associated with a gut environment that is more favorable for equol-producing bacteria. In addition, pumpkin contains vitamin E, which is thought to influence hormonal regulation [27]. Although the present study did not directly examine these mechanisms, these nutritional characteristics may partly explain the observed association between pumpkin intake and equol-producing status. Further mechanistic studies are needed to clarify whether and how pumpkin consumption contributes to equol production.

Similarly, previous reviews have reported associations between green tea or soy intake and equol production [31], which is consistent with the present findings. Green tea catechins have been suggested to support beneficial gut bacteria [26], while soybean sprouts contain relatively high levels of dietary fiber compared with other sprouts [32,33]. These dietary characteristics may contribute to a gut environment that promotes equol production, although direct mechanistic evidence in humans remains limited.

The proportion of participants experiencing PMS/PMDD symptoms in this study was higher than that reported previously among Japanese women in their twenties [11,13]. One possible explanation is that the survey period overlapped with the university examination period, which may have increased psychological stress and exacerbated symptom severity. Academic stress reportedly exacerbates PMS symptom [34].

These considerations provided the basis for examining interrelationships between equol production, dietary factors, and PMS/PMDD severity. Equol is a non-steroidal estrogen that binds estrogen receptors with lower affinity than endogenous estrogens and may exhibit both agonistic and antagonistic activity [27,35,36]. PMS/PMDD symptoms typically emerge during the luteal phase, when estrogen levels decline [9]. It is therefore plausible that equol production partially compensates for reduced estrogenic signaling during this phase; however, this hypothesis requires direct experimental validation.

Among equol producers, a positive association was observed between the frequency of black tea intake and premenstrual symptom severity. Black tea contains substantial concentrations of caffeine [37], and dietary caffeine restriction has been suggested to alleviate PMS symptoms [11]. However, given the small number of equol producers and the exploratory nature of this analysis, the present findings should be interpreted with caution and should not be taken to imply a causal relationship.

Among equol non-producers, higher consumption of konjac and konjac jelly was associated with greater PMS/PMDD severity. Konjac glucomannan modulates gut microbial composition and short-chain fatty acid production [38,39]; however, in the absence of equol-producing bacteria, such dietary modulation may not translate into symptom improvement [40]. Similarly, higher intake of pickled radish and broccoli, foods rich in insoluble fiber, was associated with greater symptom severity among non-producers. These findings may reflect differences in dietary patterns rather than direct causal effects.

Finally, equol has been recognized for its antioxidant properties. Experimental studies have demonstrated that equol exhibits stronger antioxidant activity than its precursor isoflavones and can enhance endogenous antioxidant defense systems [1,15]. Oxidative stress and neuroinflammation have been increasingly implicated in PMS pathophysiology [41]. Although oxidative stress markers were not measured in the present study, the observed associations raise the hypothesis that equol may contribute to symptom modulation through antioxidant mechanisms.

Taken together, the present findings suggest that equol-producing status may be associated with differences in dietary patterns and PMS/PMDD symptom expression in young women. While these results should be interpreted as preliminary, they support further investigation of equol as a potential biomarker and mechanistic contributor in future longitudinal and interventional studies.

## 5. Conclusions

This exploratory, cross-sectional study investigated associations among urinary equol production capacity, dietary habits, and premenstrual symptom severity in healthy young women. These findings suggest that equol production status may be associated with distinct dietary patterns and with differences in how specific food intakes relate to premenstrual symptom severity. In particular, among equol non-producers, higher intake of certain vegetables and fiber-rich foods was associated with greater symptom severity, whereas such associations were not observed among equol producers. Given the limited sample size and the cross-sectional nature of the study, causal relationships cannot be inferred with certainty. In addition, statistical analyses were exploratory, and residual confounding cannot be excluded. Nevertheless, these results suggest that urinary equol production capacity may serve as a relevant biological characteristic when considering diet-related variability in premenstrual symptoms. Future studies with larger sample sizes, longitudinal designs, and comprehensive assessment of gut microbiota and oxidative stress markers are warranted to clarify the potential role of equol in modulation of premenstrual symptoms and to evaluate its utility as a biomarker in women’s health research.

## Figures and Tables

**Table 1:** Baseline characteristics of the study population. Continuous variables are presented as means with standard deviations. Statistical comparisons were conducted using *t*-tests.

Characteristic	Median	Equol Producers	Equol Non-Producers	*p*-Value
Age (years)	19	19.2 (0.44)	19.2 (0.43)	0.836
Medical history	−	none	none	−

**Table 2:** Frequency of food intake among participants. Values with *p* < 0.05 were considered statistically significant and are marked with an asterisk (*).

Food Item	Equol Producers (*n*)	Equol Non-Producers (*n*)	*p*-Value
Bean curd	0	9	0.214
Fermented soybeans	0	21	0.180
Bean paste	2	22	0.410
Soy sauce	5	36	0.710
Soy milk	1	5	0.698
Soybean sprouts	1	3	0.396
Roasted soybean flour	3	9	0.098
Green soybeans	2	4	0.081
Fried tofu	0	5	0.382
Mushrooms	0	3	0.509
Burdock	0	9	0.214
Spinach	5	30	0.287
Broccoli	3	18	0.634
Lettuce	2	17	0.803
Cabbage	4	16	0.123
Pumpkin	3	7	0.043 *
Pickled radish	1	3	0.396
Potato	2	23	0.344
Wakame seaweed	5	31	0.331
Nori seaweed	0	5	0.382
Mekabu seaweed	0	11	0.156
Mozuku seaweed	0	1	0.710
Konjac	0	12	0.132
Coffee	1	2	0.235
Tea	3	19	0.717
Green tea	4	7	0.004 *
Beverages high in caffeine	0	6	0.331
Alcohol	0	2	0.087
Konjac jelly (present)	0	10	0.183
Konjac jelly (past)	0	5	0.652

**Table 3:** Quantities of food intake among participants categorized by equol production status. Values with *p* < 0.05 were considered statistically significant and are marked with an asterisk (*).

Food Item	Equol Producers (n)	Equol Non-Producers (n)	*p*-Value
Bean curd	2	24	0.832
Fermented soybeans	0	29	0.934
Bean paste	3	32	0.136
Soy sauce	4	33	0.150
Soy milk	1	16	0.321
Soybean sprouts	2	30	0.043 *
Roasted soybean flour	3	12	0.228
Green soybeans	1	21	0.123
Fried tofu	1	15	0.375
Mushrooms	3	23	0.062
Burdock	2	26	0.178
Spinach	3	25	0.737
Broccoli	5	31	0.331
Lettuce	4	36	0.089
Cabbage	4	34	0.509
Pumpkin	2	21	0.048 *
Pickled radish	1	16	0.321
Potato	3	32	0.136
Wakame seaweed	3	23	0.062
Nori seaweed	4	24	0.501
Mekabu seaweed	4	25	0.573
Mozuku seaweed	0	11	0.156
Konjac	5	12	0.132
Coffee	1	19	0.188
Tea	1	3	0.396
Green tea	4	26	0.652
Beverages high in caffeine	5	32	0.382
Alcohol	1	6	0.832
Konjac jelly (present)	0	8	0.214
Konjac jelly (past)	2	9	0.455

**Table 4:** Numbers of participants stratified by equol-producing status and PMS/PMDD severity.

PMS/PMDD	No-Mild		Moderate		Severe	
Equol production ability	−	+	−	+	−	+
N	11	1	25	3	1	1

**Table 5:** Unadjusted and adjusted logistic regression model using generalized estimating equations to account for equol-producing capacity.

	Unadjusted OR (95%CI)	*p* Value	Adjusted OR (95%CI)	*p* Value
Age	0.479 (0.097–2.380)	0.368	0.496 (0.099–2.49)	0.394
PMS severity	0.750 (0.233–2.420)	0.63	0.804 (0.246–2.63)	0.718

Abbreviations: OR, odds ratio; CI, confidence interval.

**Table 6:** Statistical association between PMS/PMDD severity and frequency of dietary intake among equol producers. *p* < 0.05 was considered significant and is marked with an asterisk (*).

Food Item	Moderate–Severe (n)	None–Mild (n)	*p*-Value
Bean curd	2	1	0.710
Fermented soybeans	2	2	0.362
Bean paste	2	2	0.362
Soy sauce	3	2	1.000
Soy milk	0	1	0.171
Soybean sprouts	0	1	0.171
Roasted soybean flour	2	1	0.710
Green soybeans	2	0	0.137
Fried tofu	0	0	1.000
Mushrooms	0	0	1.000
Burdock	0	0	1.000
Spinach	3	2	1.000
Broccoli	2	1	0.710
Lettuce	1	1	0.710
Cabbage	0	1	0.171
Pumpkin	1	2	0.137
Pickled radish	1	0	0.362
Potato	1	1	0.710
Wakame seaweed	3	2	1.000
Nori seaweed	0	0	1.000
Mekabu seaweed	0	0	1.000
Mozuku seaweed	0	0	1.000
Konjac	0	0	1.000
Coffee	1	0	0.362
Black Tea	3	1	0.026 *
Green tea	2	2	0.362
Beverages high in caffeine	0	0	1.000
Alcohol	0	0	1.000
Konjac jelly (present)	0	0	1.000
Konjac jelly (past)	0	1	0.362

**Table 7:** Statistical association between PMS/PMDD severity and quantity of dietary intake among equol producers.

Food Item	Moderate–Severe (n)	None–Mild (n)	*p*-Value
Bean curd	2	1	0.710
Fermented soybeans	2	2	0.362
Bean paste	2	1	0.710
Soy sauce	2	2	0.362
Soy milk	1	0	0.362
Soybean sprouts	1	1	0.710
Roasted soybean flour	2	1	0.710
Green soybeans	1	0	0.362
Fried tofu	1	0	0.362
Mushrooms	2	1	0.710
Burdock	1	1	0.710
Spinach	1	2	0.137
Broccoli	3	2	1.000
Lettuce	2	2	0.362
Cabbage	3	2	1.000
Pumpkin	1	1	0.710
Pickled radish	1	0	0.362
Potato	2	1	0.710
Wakame seaweed	3	1	0.171
Nori seaweed	2	2	0.362
Mekabu seaweed	0	0	1.000
Mozuku seaweed	1	0	0.362
Konjac	1	0	0.362
Coffee	1	0	0.362
Black Tea	3	1	0.171
Green tea	3	2	1.000
Beverages high in caffeine	1	0	0.362
Alcohol	0	0	1.000
Konjac jelly (present)	1	1	0.710
Konjac jelly (past)	unknown	unknown	―

**Table 8:** Statistical association between PMS/PMDD severity and frequency of dietary intake among non-equol producers. Significance determined at *p* < 0.05 and marked with an asterisk (*).

Food Item	Moderate–Severe (n)	None–Mild (n)	*p*-Value
Bean curd	4	4	0.465
Fermented soybeans	9	11	0.401
Bean paste	8	11	0.204
Soy sauce	14	21	0.419
Soy milk	3	2	0.127
Soybean sprouts	2	1	0.303
Roasted soybean flour	3	5	0.694
Green soybeans	2	2	0.629
Fried tofu	2	3	0.957
Mushrooms	1	2	0.837
Burdock	5	4	0.237
Spinach	9	20	0.05
Broccoli	10	8	0.041 *
Lettuce	9	7	0.056
Cabbage	5	8	0.419
Pumpkin	4	3	0.270
Pickled radish	2	1	0.303
Potato	9	13	0.756
Wakame seaweed	13	17	0.222
Nori seaweed	3	6	0.297
Mekabu seaweed	5	6	0.592
Mozuku seaweed	0	1	0.410
Konjac	3	2	0.297
Coffee	2	3	0.957
Black Tea	4	9	0.453
Green tea	8	9	0.342
Beverages high in caffeine	2	4	0.760
Alcohol	0	2	0.246
Konjac jelly (present)	4	6	0.933
Konjac jelly (past)	6	5	0.292

**Table 9:** Statistical association between PMS/PMDD severity and quantity of dietary intake among non-equol producers. Values with *p* < 0.05 indicate statistical significance and are marked with an asterisk (*).

Food Item	Moderate–Severe (n)	None–Mild (n)	*p*-Value
Bean curd	11	12	0.144
Fermented soybeans	9	19	0.121
Bean paste	13	18	0.351
Soy sauce	13	19	0.546
Soy milk	6	9	0.908
Soybean sprouts	13	16	0.137
Roasted soybean flour	5	6	0.592
Green soybeans	10	10	0.127
Fried tofu	5	9	0.756
Mushrooms	9	13	0.756
Burdock	12	13	0.091
Spinach	10	14	0.629
Broccoli	13	17	0.222
Lettuce	14	21	0.419
Cabbage	13	20	0.837
Pumpkin	9	11	0.401
Pickled radish	9	6	0.029 *
Potato	12	19	0.957
Wakame seaweed	10	13	0.453
Nori seaweed	11	13	0.227
Mekabu seaweed	4	6	0.993
Mozuku seaweed	4	7	0.837
Konjac	10	8	0.041 *
Coffee	2	1	0.303
Black Tea	8	15	0.837
Green tea	12	19	0.957
Beverages high in caffeine	3	3	0.541
Alcohol	2	5	0.533
Konjac jelly (present)	6	3	0.049 *
Konjac jelly (past)	unknown	unknown	

## Data Availability

Datasets generated and/or analyzed during this study are available from the corresponding author upon request.

## Supplementary Materials

The following supporting information can be downloaded at: https://www.mdpi.com/article/10.3390/metabo16010055/s1, Figure S1: Chromatograms of two blank samples; Figure S2: Calibration curve of Equol; Table S1: PMS/PMDD Questionnaire and Diagnostic Criteria; Table S2: Intra-day accuracy and precision; Table S3: Inter-day precision; Table S4: Extract Stability.

## Abbreviations

The following abbreviations are used in this manuscript:

LC-MS/MS	Liquid Chromatography–Tandem Mass Spectrometry
PMDD	Premenstrual Dysphoric Disorder
PMS	Premenstrual Syndrome
ROS	Reactive Oxygen Species
HbA1c	Hemoglobin A1c
