# Supplementary material for: Urinary Equol Production Capacity, Dietary Habits, and Premenstrual Symptom Severity in Healthy Young Japanese Women
Source: Metabolites. 2026 Jan 8;16(1):55. doi: 10.3390/metabo16010055 (PMC12844494; doi:10.3390/metabo16010055)
Supplement: Supplementary file 1 [file metabolites-16-00055-s001.zip › metabolites-4040730-supplementary.pdf]

Supplementary information

Urinary Equol Production Capacity, Dietary Habits, and Premenstrual Symptom Severity in Healthy Young Japanese Women

Nanae Kada-Kondo [1,2], Natsuka Kimura [3], Kurea Isobe [2], Akari Kaida [2], Saki Ota [2], Akari Fujita [2], Yuu Haraki [2], Ryozo Nagai [4] and Kenichi Aizawa [3,*]

1 Department of Food Science, Faculty of Home Economics, Otsuma Women's University
2 Department of Nutrition and Dietetics, Faculty of Home Economics, Kamakura Women's University
3 Department of Translational Research, Clinical Research Center, Jichi Medical University Hospital,
Shimotsuke, Tochigi 329-0498, Japan
4 Jichi Medical University, Shimotsuke 329-0498, Japan.
* Correspondence: aizawa@jichi.ac.

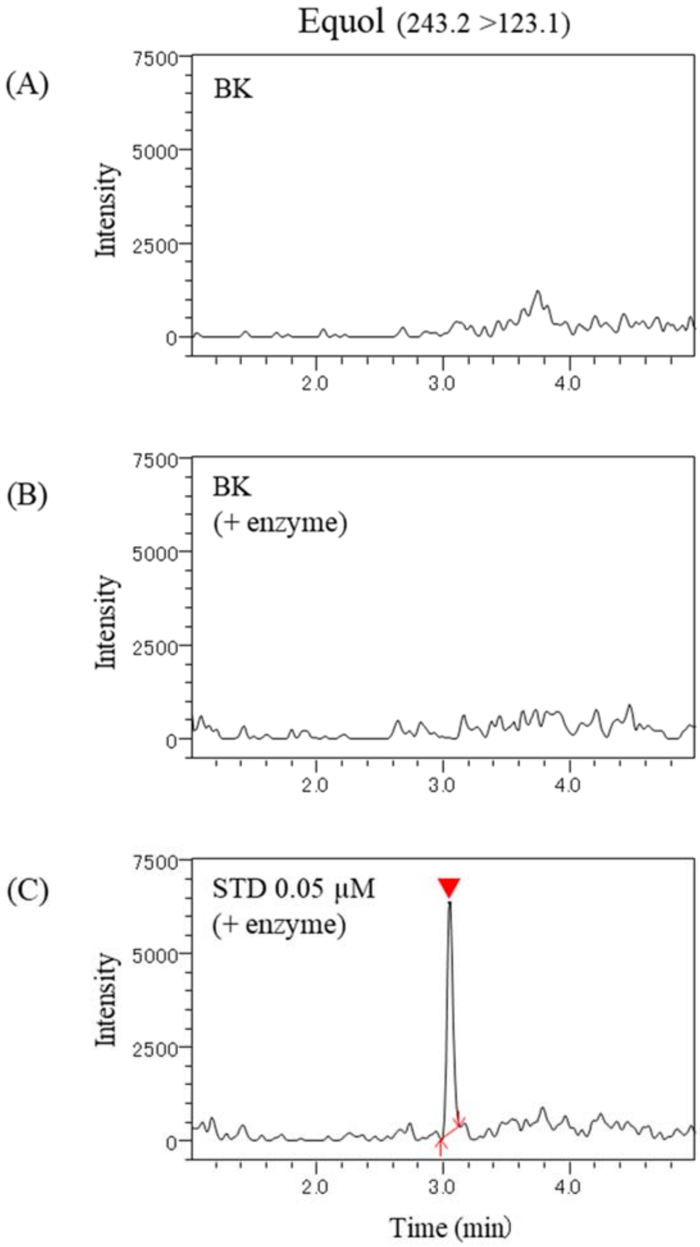

Supplementary Figure S1. Chromatograms of two blank samples.

(A) without enzyme and (B) enzyme added with (C) the chromatogram of the LOQ (Limit of Quantification). No peaks interfered with identification of EQ.

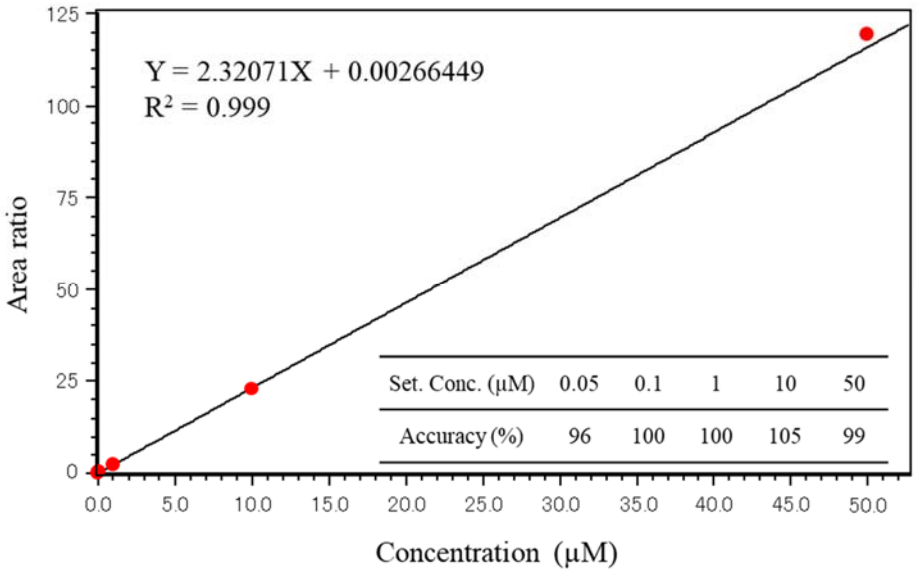

Supplementary Figure S2. Calibration curve of Equol.

Values of the Y-axis were calculated using the ratio of the equol area value to the equol internal standard area value. This assay demonstrated high sensitivity and specificity, with a lower quantification of 0.05 μM.

Supplementary Table S1. PMS/PMDD Questionnaire and Diagnostic Criteria. From a questionnaire consisting of 11 symptom items and 5 items assessing impact on daily life, the severity of PMS/PMDD symptoms was recorded and evaluated based on the total score.

1. Symptom items: Do you experience any of the following symptoms during the week preceding menstruation and during menstruation?

I. Symptom items	Not at all	Mild	Moderate	Severe
1. Depressed mood / feeling down				
2. Emotional instability / mood swings				
3. Anxiety or excessive worry				
4. Irritability / feeling easily angered				
5. a. Decreased motivation<br>b. Difficulty concentrating<br>c. Feeling confused or unable to organize thoughts				
6. Insomnia				
7. Fatigue / general malaise				
8. Increased appetite or food cravings				
9. a. Insomia<br>b. Excessive sleepness				
10. Inability to control emotions				
11. Physical symptoms<br>(Breast tenderness / pain, Headache,<br>Muscle pain, Joint pain, Abdominal bloating<br>Edema, Weight gain)				

2. Impact of daily life: How much do these symptoms interfere with your daily life?

II. Impact on daily life	Not at all	Mild	Moderate	Severe
A. Work or study performance				
B. Interpersonal relationships at school or work				
C. Relationships with my family and partner				
D. Hobbies and leisure activities				
D. Household chores				

Diagnosis Criteria (the combined score of 1 and 2)

Category	Criteria
**PMDD**	• At least five of items #1–11 rated as moderate to severe, including at least one of items #1–4 rated as severe; and<br>• At least one of items A–D rated as severe.
**Severe PMS**	• At least five of items #1–11 rated as moderate to severe, including at least one of items #1–4 rated as moderate; and<br>• At least one of items A–D rated as severe.
**Moderate PMS**	• At least five of items #1–11 rated as moderate to severe, including at least one of items #1–4 rated as moderate; and<br>• At least one of items A–D rated as moderate.
**No–Mild PMS**	• Fewer than five symptom items (#1–11) rated as moderate to severe; or<br>• All impact items (A–D) rated as none or mild.

Supplementary Table S2. Intra-day accuracy and precision

Set Conc. (μM)	QC samples	Conc. (μM)	Accuracy (%)	CV (%)
0.2	1	0.19	94	2
	2	0.19	96	6
	3	0.18	90	3
	4	0.19	97	1
	5	0.18	91	3
20	1	20.5	102	6
	2	18.6	93	9
	3	18.3	91	4
	4	18.7	94	5
	5	19.2	96	3
40	1	40.5	101	6
	2	39.8	100	1
	3	40.0	100	5
	4	38.5	96	7
	5	40.5	101	6

Supplementary Table S3. Inter-day precision

		**Conc. (µM)**	**CV (%)**
QC samples	Low	0.1	11
	Middle	1.0	6
	High	9.9	5
Urine samples	1	7.1	7
	2	N.A.	-
	3	N.A.	-
	4	0.4	12

Supplementary Table S4. Extract Stability

		Area ratio				CV (%)
		0 h	12 h	24 h	Avg.	
QC samples	Low	0.510	0.482	0.478	0.490	4
	Middle	3.826	4.103	4.489	4.140	8
	High	42.32	38.55	42.96	41.28	6
Urine samples	1	30.75	30.00	28.14	29.63	5
	2	-	-	-	-	-
	3	-	-	-	-	-
	4	1.69	1.83	1.51	1.68	10
